# Biomass Production Potential of a Wastewater Alga *Chlorella vulgaris* ARC 1 under Elevated Levels of CO_2_ and Temperature

**DOI:** 10.3390/ijms10020518

**Published:** 2009-02-05

**Authors:** Senthil Chinnasamy, Balasubramanian Ramakrishnan, Ashish Bhatnagar, Keshav C. Das

**Affiliations:** 1 Department of Biological and Agricultural Engineering, The University of Georgia, Athens, GA 30602, USA; E-mails: bhatnagarashis@gmail.com (A.B.); kdas@engr.uga.edu (K.C.D.); 2 Laboratory of Soil Microbiology, Division of Soil Science and Microbiology, Central Rice Research Institute, Cuttack 753006, Orissa, India; E-mail: ramakrishnanbala@yahoo.com

**Keywords:** Biomass, carbonic anhydrase, Chlorella, CO_2_, ^14^C uptake, microalgae, temperature

## Abstract

The growth response of *Chlorella vulgaris* was studied under varying concentrations of carbon dioxide (ranging from 0.036 to 20%) and temperature (30, 40 and 50°C). The highest chlorophyll concentration (11 μg mL^–1^) and biomass (210 μg mL^–1^), which were 60 and 20 times more than that of *C. vulgaris* at ambient CO_2_ (0.036%), were recorded at 6% CO_2_ level. At 16% CO_2_ level, the concentrations of chlorophyll and biomass values were comparable to those at ambient CO_2_ but further increases in the CO_2_ level decreased both of them. Results showed that the optimum temperature for biomass production was 30°C under elevated CO_2_ (6%). Although increases in temperature above 30°C resulted in concomitant decrease in growth response, their adverse effects were significantly subdued at elevated CO_2_. There were also differential responses of the alga, assessed in terms of NaH^14^CO_3_ uptake and carbonic anhydrase activity, to increases in temperature at elevated CO_2_. The results indicated that *Chlorella vulgaris* grew better at elevated CO_2_ level at 30°C, albeit with lesser efficiencies at higher temperatures.

## 1. Introduction

Carbon dioxide, the most plentiful of trace gases responsible for the greenhouse effect, is steadily increasing due to anthropogenic activities [1,2]. More than 170 countries have ratified the 1997 Kyoto Protocol of the United Nations to make efforts to reduce greenhouse gases by 5.2% on the basis of the emissions in 1990 [3]. Biological CO_2_ mitigation appears an attractive strategy as it produces biomass based energy in the process of CO_2_ fixation through photosynthesis [1,4,5].

CO_2_ mitigation by plants contributes only to 3–6% of fossil fuel emissions [6]. Amongst all phototrophic organisms, aquatic microbial oxygenic phototrophs (cyanobacteria and microalgae) are special inasmuch as they grow fast, perform 10–50 times more efficient photosynthesis than plants, and require lesser energy and labour than the cultivation of crops [7]. In recent times, microalgae have been promoted for CO_2_ mitigation as they can be grown in wastewaters and capture CO_2_ from high-CO_2_ streams such as flue and flaring gases with CO_2_ contents ranging from 5–15% [8]. Further, they do not pose any risk to drive up food prices and exacerbate CO_2_ release through the forced clearing of natural ecosystems [9].

Earlier it was assumed that microalgae were susceptible to high CO_2_ concentrations. However, some microalgae are now reported to grow rapidly even at very high CO_2_ concentrations [10–12]. Extreme CO_2_ concentrations – about 1,000 times higher than the ambient level (0.036%), can be easily fixed to biomass by the unicellular green algae [13]. Therefore, algae that tolerate high CO_2_ levels may be identified. It is also important to find the upper temperature limits of algal growth so that the flue gases might need lesser cooling. Most of the studies on CO_2_ mitigation provide little information on the physiological responses of algae grown under elevated CO_2_ and increased temperature conditions. Therefore, this study aimed at determining the effects of elevated CO_2_ concentration under three different temperature conditions on a unicellular green alga *Chlorella vulgaris* ARC 1. The response was monitored in terms of changes in biomass, biochemical composition (pigments, carbohydrates and proteins), the rates of C^14^ uptake and the activity of carbonic anhydrase.

## 2. Results

### 2.1. Growth response to increasing concentrations of CO_2_

**Figure 1:**
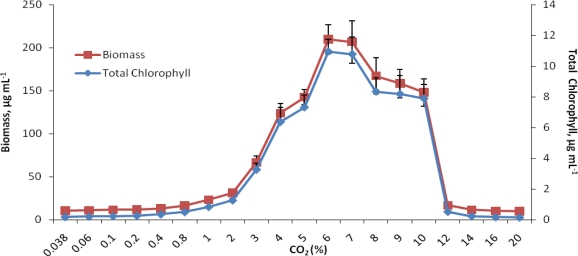
Growth response of *Chlorella vulgaris* ARC 1 at graded CO_2_ concentrations. Data points represent mean values of triplicates and error bars are standard deviations. C.D. at 5% for chlorophyll and biomass is 0.343 and 7.860, respectively.

The highest chlorophyll content (11 μg mL^−1^) and dry weight (210 μg mL^−1^) of *C. vulgaris*, which were 60 and 20 times more than that of alga under ambient CO_2_, were recorded at 6% CO_2_ level (Figure 1).

**Figure 2:**
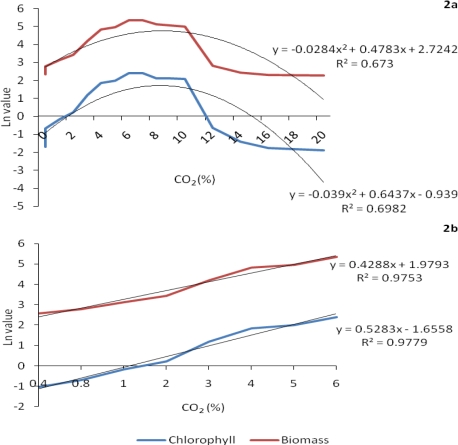
Trends and predictability of chlorophyll and biomass (in natural log) when grown at elevated CO_2_ concentration. Figure 2a, incorporating all levels of CO_2_ up to 20%, shows a sigmoidal curve fitting polynomial equation while 2b shows a consistent log linear increase in both parameters till a rise of CO_2_ to 6% level with highly significant coefficient of determination.

Interestingly, the alga showed a sharp increase in chlorophyll synthesis and biomass production when the CO_2_ concentration was raised from 1% to 6%. So much so that when log transformed, it could fit linear regression equations with R^2^ being more than 0.97, although overall growth from ambient to the 20% CO_2_ level showed a bell shaped curve (Figures 2a and 2b). A maximum increase of 158% in chlorophyll content and 114% in dry weight was observed, especially when the CO_2_ level was raised from 2 to 3%. Growth reached a plateau between 6 to 12% CO_2_ and then declined sharply, yet at 16% CO_2_ level, the values for chlorophyll and biomass were comparable to those obtained at ambient CO_2_. In the presence of ambient and 6% CO_2_ concentration, *C. vulgaris* was further examined for its growth performance and ability to synthesize cell constituents at 30, 40 and 50 °C.

### 2.2. Growth at elevated CO_2_ and temperatures

**Figure 3:**
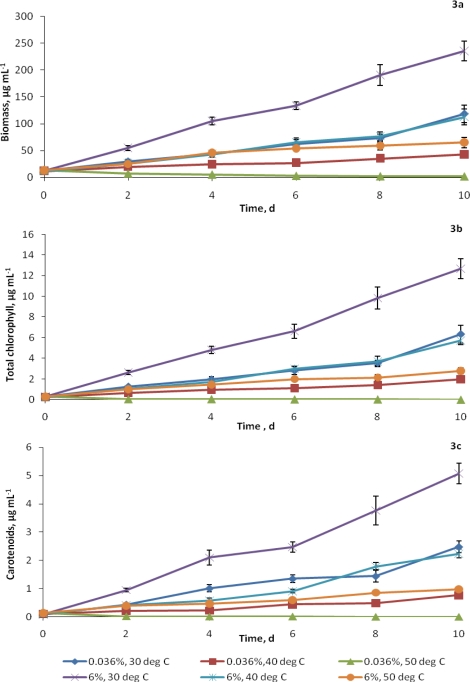
Effect of elevated CO_2_ and temperature on (a) biomass, (b) total chlorophyll and (c) carotenoid contents (μg mL^−1^) of *C. vulgaris* Data points represent mean values of triplicates and error bars are standard deviations. C.D. at 5% level [CO_2_ concentration x Temperature x Days] for biomass, total chlorophyll and carotenoids is 5.720, 0.246 and 0.136, respectively.

A time course dependent increase in biomass production at both ambient and elevated CO_2_ levels was observed with this algal strain (Figure 3a). The alga grew vigorously at elevated CO_2_ (6%), resulting in a sharp and significant increase in the biomass content that was observed even on the 2^nd^ day of incubation. The optimum temperature for both levels of CO_2_ tested for this alga was 30°C.

The biomass production of this algal culture grown at 6% CO_2_, 30°C temperature and a light intensity of 47 μmol photons m^−2^ s^−1^ was about 99%, compared to that at ambient CO_2_, after 10 days of incubation. Also, the culture grown at ambient level of CO_2_ at 30^°^C recorded 879% increase in biomass production against 1846% of that at 6% CO_2_. The adverse effect of high temperature was, however, significantly subdued at 6% CO_2_ where the reduction at 50°C was about 3.5 times as compared to 61.5 times of the culture grown under ambient CO_2_ level.

Temperature affected the growth rate and biomass productivity (Table 1). The maximum specific growth rate (μ = 0.222 d^−1^) was supported by elevated CO_2_ at 30°C, while ambient CO_2_ at 30°C as well as elevated CO_2_ at 40°C had almost the same specific growth rate (0.128 and 0.136 d^−1^, respectively). There was no growth at 50°C and ambient CO_2_ level.

### 2.3. Synthesis of cellular constituents at elevated CO_2_ and temperatures

The overall trend was a general increased response in all cell constituents *vis a vis* biomass over time. The chlorophyll content increased with time, irrespective of the rise in temperature and increase in CO_2_ level, except under ambient CO_2_ and 50°C conditions, where the alga showed signs of bleaching within 2 days of incubation. At ambient CO_2_ level, the chlorophyll content increased by 22- fold after 10 days of incubation at 30°C, as compared to only a 7-fold of increase at 40°C. But under elevated CO_2_ (6%), the increase was about 44 and 9 times in cultures grown at 30 and 40°C, respectively (Figure 3b). Further increase in temperature from 40 to 50°C resulted in a decrease in the chlorophyll content. Rates of doubling of total chlorophyll also showed a trend similar to that of biomass (Table 1).

On 10^th^ day of incubation, the carotenoid content of alga at elevated CO_2_ was 2.04 fold higher than that of ambient CO_2_ (Figure 3c). This trend was also reflected in the time dependent increase of carotenoid content at each temperature tested, except at ambient CO_2_ at 50°C, but the magnitude of increase reduced with high temperature.

**Figure 4:**
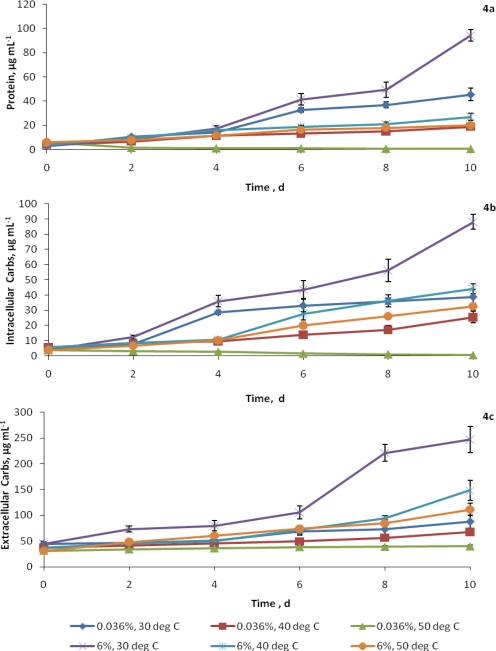
Effect of elevated CO_2_ and temperature on (a) protein, (b) intracellular and (c) extracellular carbohydrates (μg mL^−1^) of *C. vulgaris.* Data points represent mean values of triplicates and error bars are standard deviations. C.D. at 5% level [CO_2_ concentration x Temperature x Days] for protein, intracellular carbohydrates and extracellular carbohydrates is 1.870, 2.501 and 5.821, respectively.

Total amount of proteins synthesized by the alga was maximum at 30°C and 6% CO_2_ level and the increase was significant, compared to that of ambient CO_2_ (Figure 4a). Proteins produced in presence of elevated CO_2_ (6%) were more than double the quantity of what was produced at ambient CO_2_ on the 10^th^ day of incubation. The elevated CO_2_ level appeared to have a moderating effect at 50°C as the protein content increased by about 3.3 fold but at the same temperature (50°C), a 9 fold reduction occurred when the CO_2_ level was ambient. Higher CO_2_ level significantly increased the protein content of alga at all treatments on 2^nd^ day of growth itself.

Optimum temperature for intracellular carbohydrate production was 30°C and 6% CO_2_ enhanced this effect by a factor of 2.27 on the 10^th^ day of incubation (Figure 4b). At 30°C and ambient CO_2_, the increase in extracellular carbohydrate on the 10^th^ day was 1.9 times, compared to that of intracellular carbohydrate concentration, but at 6% CO_2_, it was enhanced by 5.6 times (Figure 4c). The same trend was seen at 40 and 50°C also, where the increase was 1.8 and 1.3 times under ambient CO_2_ as compared to 4 and 3.5 times respectively, at elevated CO_2_. Extracellular carbohydrate production was significantly higher at 6% CO_2_ than the ambient level at temperatures tested. Unlike other cell constituents, extracellular carbohydrates remained constant, instead of decreasing at 50°C under ambient CO_2_.

### 2.4. ^14^C uptake and carbonic anhydrase activity in response to elevated CO_2_ and temperatures

The algal strain grown at 30°C and ambient CO_2_ concentration fixed 7.833 μmol of NaH^14^CO_3_ compared to 6.033 μmol, a decline of 29% under elevated CO_2_ (Table 2). At the ambient level of CO_2_, an increase in the temperature to 40°C further enhanced ^14^CO_2_ uptake by 23%. On the contrary, the cells were able to take up only 3.413 μmol of NaH^14^CO_3_ at the same temperature (40°C) and elevated CO_2_.

Further increase in temperature to 50°C did not significantly alter the ^14^CO_2_ uptake at 6% CO_2_ while it showed almost no uptake under ambient CO_2_. The end point study on the intracellular carbonic anhydrase activity showed that an increase in temperature significantly reduced the enzyme activity but any increase in CO_2_ level appeared to have a synergistic effect. At 30°C, the carbonic anhydrase activity was maximum at ambient CO_2_ while it was significantly reduced by 46% at elevated CO_2_ (Table 2). Increase in temperature to 40°C under ambient CO_2_ reduced the enzyme activity by 17%, but the reduction was 23% at elevated CO_2_. Although carbonic anhydrase activity was significantly reduced at elevated CO_2_ and 30°C temperature, a moderating effect of increased level of CO_2_ was noted at 40 and 50°C. The response of the enzyme to increase in temperature was substantially subdued at 6% CO_2_ level.

## 3. Discussion

Algae play a vital role in the biogeochemical cycling of carbon. Although many algae are able to utilise different sources of organic carbon, CO_2_ is the main source of carbon for the majority of them under illuminated conditions. Some microalgae are reported to grow very rapidly at CO_2_ concentrations of more than 40% [11]. *Chlorella vulgaris* ARC 1 used in the present study grew very well and showed significant gains in chlorophyll content and biomass up to 6% CO_2_ level. It maintained the superiority over ambient CO_2_ level even when the CO_2_ was raised up to 16%. Kodama *et al*. reported that *Chlorella littorale* - a marine alga - performed better at 30°C, pH 4 and 20% CO_2_ concentration and another unicellular marine alga could grow rapidly even at 60% CO_2_ level [10]. Xia and Gao found that *Chlorella pyrenoidosa* showed improved growth at 186 μmol CO_2_ L^−1^ while *Chlamydomonas reinhardtii* did not show significant improvement in growth at 186 over 21 μmol CO_2_ L^−1^ [14]. The existence of wide variation in the response of algae to the elevated levels of CO_2_, also observed in our preliminary study (data not shown), suggests the possibility for the isolation of such naturally occurring algal forms that may scavenge CO_2_ efficiently. It also suggests of opportunities for the manipulation of such organisms to produce utility molecules.

Growth response of *C. vulgaris* in terms of biomass and total chlorophyll showed similar pattern. However, their values could not corroborate growth rate since chlorophyll *b* does not behave in correspondence to the growth (Table 1). Environmental conditions also modify Chl *a*/Chl *b* ratio [14]. *Chlorella kessleri* has been shown to have a maximum specific growth rate of 0.267 d^−1^ when cultivated with 6% CO_2_ while *C. vulgaris* in the present study had a comparable rate of 0.222 d^−1^ at the same level of CO_2_ [12].

In general there was an increase in proteins from 39.8 to 40.8% and carbohydrates from 33 to 37.8% under ambient and elevated CO_2_ respectively at 30°C. However the amount of lipids reduced from 12.8 to 7% and ash and nucleic acids remained constant at ∼14.4% (data not shown). Chiu *et al*. also observed a decrease in the lipid content of *Nannochloropsis oculata* at elevated CO_2_ levels of 5, 10 and 15% [15].

Temperature effectively regulates various metabolic processes and the interaction between CO_2_ concentration and temperature is bound to reflect in growth and biomass production by algae. The tested algal strain performed better at 30°C and the growth improved significantly when the CO_2_ level was raised from ambient to 6%. Sakai *et al*. reported isolates of a *Chlorella* sp. from hot springs in Japan that grew up to 42°C when bubbled with air containing 40% CO_2_ [16]. The results of the present study show that the ambient CO_2_ could not support growth of *C. vulgaris* at 50°C but higher CO_2_ (6%) recorded significant growth. The general notion is that increase in the algal performance at higher levels of CO_2_ may be because of the enhanced availability of dissolved CO_2_ and the toxicity of a very high CO_2_ concentration is due to the lowering of pH. We observed that headspace content of 6% and 20% CO_2_, lowered the pH from initial 7.5 to 6.9 and 6.3, respectively and the growth of *C. vulgaris* raised it to 8 and 6.8, respectively after 10 days of incubation. Since the batch cultures in the present study were grown under continuous supply of CO_2_-air mixture keeping light and other nutrients uniform; thus the observed influence on various growth parameters could be attributed to the increased availability of CO_2_ at elevated CO_2_ level. The moderating effect of elevated CO_2_ level on the influence of higher temperature (even at 50°C) on algal growth suggested that although increases in atmospheric CO_2_ concentration were often associated with rise in temperature, the adverse effect of increased temperature on algal growth might be substantially reduced by the enhanced CO_2_.

The positive effects of higher level (6%) of CO_2_ were observed on many cellular constituents of the algal strain tested in the present study. Increase in the pigment content can complement the increased demand of energy for reducing more CO_2_ to carbohydrates. The presence of an efficient light harvesting system at higher level of CO_2_ enhances the photosynthetic activity, which in turn generates more reductant and energy donor. The microalga *Chlorella vulgaris* is known to produce pharmaceutically important carotenoids: canthaxanthin and astaxanthin [17]. Increased production of carotenoids in presence of higher amounts CO_2_ might add to the economic utility of this algal strain.

It is demonstrated that carbonic anhydrase is essential for photosynthetic utilisation of inorganic carbon at low external CO_2_ concentrations and alkaline pH [18]. Its activity decreases or disappears in eukaryotic microalgae under air enriched with 1–5% CO_2_ [19]. However, Xia and Gao reported that the susceptibility levels are species dependent [14]. In the present study, the ^14^CO_2_ uptake was significantly more in the cells pre-incubated at ambient CO_2_ than at elevated (6%) CO_2_ suggesting increased photosynthetic rate. Increase in temperature enhances the process of photorespiration more markedly at low CO_2_ levels thus causing depletion of intracellular CO_2_ and other carbon reserves. This probably lead to the significant increase in the ^14^CO_2_ uptake of *C. vulgaris* at ambient CO_2_ when the temperature was raised from 30 to 40°C. Interestingly temperature increases (40 and 50°C) at ambient CO_2_ level significantly reduced the algal growth performance but 6% CO_2_ concentration could effectively restrain this reduction. Hanagata *et al*. also reported induction of temperature endurance in *Chlorella* at 20% CO_2_ level, largely due to enhanced photosynthetic activity [20]. On the contrary, DeLucia *et al*. found that the rate of photosynthesis increased markedly only for a short period immediately after the exposure to high levels of CO_2_ and slowed down subsequently [21]. Although algae are able to utilise CO_2_, carbonate (CO_3_^−^) and bicarbonate (HCO_3_^−^), the most preferred source is CO_2_ under normal conditions. The transport of CO_2_ across the plasma membrane is energy dependent and its accumulation in the cells is done in the form of HCO_3_^−^. The later is converted to CO_2_ by carbonic anhydrase located in the pyrenoids present in the chloroplasts [22]. The results of the present study showed that the activity of carbonic anhydrase at 30°C reduced by 46% at elevated (6%) CO_2_ level *vis a vis* ambient CO_2_, while Xia and Gao observed 45.5% and ∼36% reduction in extracellular carbonic anhydrase activity in *Chlamydomonas reinhardtii* and *Chlorella pyrenoidosa,* respectively at just 187 μmol L^−1^ CO_2_ as against 3 μmol L^−1^ of CO_2_ at 25°C [14]. Our results suggest that the carbonic anhydrase of *Chlorella vulgaris* had far better tolerance for high CO_2_ levels.

The attribute of showing better performance at higher CO_2_ level is effectively employed to develop the concept of algae mediated removal of nutrients from municipal and industrial wastewaters. FitzGerald and Rohlich reported that since carbon becomes limiting before nitrogen, there is always a possibility of enhancing the removal of nitrogen by bubbling CO_2_ through sewage [23]. This principle has since been utilised in increasing the efficiency of high rate oxidation ponds. Likewise, microalgae having high affinity for polyvalent metals are effectively used to reduce the concentration of heavy metals present in water and wastewater [24]. High CO_2_ and high temperature create a stress situation that may be tolerated by selected organisms only. This reduces competition and encourages bulking or dominance of the tolerant form(s). Hence, an increased potential for growth under such a situation will be an environmentally as well as economically attractive attribute for the utilisation of algae.

## 4. Conclusions

*C. vulgaris* in the present study could produce biomass at high CO_2_-high temperature condition. Based on its biomass generation ability it could fix 18.3 and 38.4 mg CO_2_ L^−1^ d^−1^ at ambient and elevated CO_2_ (6%), respectively under 47 μmol m^−2^ s^−1^ photon density. Since natural sunlight provides four times more photon density than the experimental conditions, it is expected that ∼120 ton CO_2_ ha^−1^ year^−1^ may be fixed by the alga using 6% CO_2_. However, the interpretation is limited by the fact that the estimation disregards efficiency of CO_2_ utilisation. The process of CO_2_ mitigation may be economized by integrating it with production of algal biomass for biofuel along with value added product(s) while utilizing waste streams as source of nutrients.

## 5. Experimental Section

### 5.1. Experimental organism

The algal strain, namely *Chlorella vulgaris* ARC 1, was selected after a preliminary screening using different algal species. This green alga was originally isolated from Nehru Vihar Oxidation Pond System at Delhi (India) and was obtained from the Centre for Conservation and Utilisation of Blue Green Algae, Indian Agricultural Research Institute, New Delhi, India. It was very common in the wastewater where the BOD_5_ levels varied from 55–720 at the inlet and 9–60 mg L^−1^ at the outlet [25]. The strain was maintained in the complete BG11 medium supplemented with 1.5% sodium nitrate and was exposed to an irradiance of 27 μmol photons m^−2^ s^−1^, a light/dark-cycle of 12/12 h and a temperature of 28 ± 1°C in a fully automated growth room [26].

### 5.2. Growth response to varied carbon dioxide concentrations

The *C. vulgaris* was grown in 100 mL of BG11 medium in air-tight bottles of 500 mL capacity. The headspace atmosphere was altered with graded concentrations of CO_2_ ranging from 0.03 to 20%, by withdrawing the air and then injecting appropriate quantity of CO_2_ into the bottles after opening the lid every day, using commercial grade CO_2_ (99.97% v/v with less than 10 μL L^−1^ CO). Each treatment had three replicates. The incubation vessels were tightly closed with rubber stoppers to prevent leakage. The biomass and total chlorophyll content were determined after 10 days of incubation in the growth room under conditions mentioned above and reported in terms of amount (μg) per unit volume (mL) of the culture.

### 5.3. Experimental CO_2_ chamber set-up

**Figure 5:**
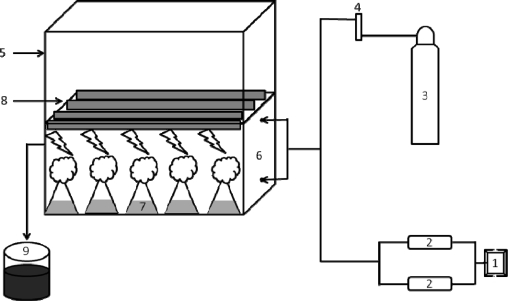
Diagram of the experimental CO_2_ chamber (1) Timer (2) Air circulation pumps (3) CO_2_ gas cylinder (4) Flow meter (5) Culture rack (6) CO_2_ chamber (7) Algal culture (8) Light source (9) Saturated KOH.

The open top chamber as described earlier by Rogers *et al*. served as the prototype and was modified into a semi-closed system [27]. Cubical Plexiglass chambers (38.6 cm height, 55 cm width, 123.1 cm length, 0.2613 m^3^ volume) fitted with four cool day light fluorescent tube lamps, a fan and thermometer were constructed by modifying a culture rack within the culture room having facilities to control temperature and duration of illumination (Figure 5).

The front portion of each chamber was provided with three partly overlapping sliding doors, which were properly closed and sealed while the experiment was in progress. The CO_2_ chamber had two entry points for CO_2_-air mixture and three exit points to avoid the build up of positive pressure inside the chamber. The commercial grade CO_2_ was released through a regulator @ 4 kg cm^−2^ and allowed to pass through a flow meter @ 500 mL min^−1^ to maintain the required concentration (6% CO_2_) inside the chamber. The air with ambient level of CO_2_ was sucked through an air pump, using a PVC tubing from outside the growth room. The CO_2_ and air were mixed using a “Y” tube and pumped into the chamber through two entry points, one at the top and another at the base of the chamber. Pumping of air was ensured by employing two oil free air pumps programmed to work alternately round the clock and the concentration was regularly monitored with the help of Ametek Carbon dioxide Analyser CD-3A. A 12 h light/dark cycle was maintained for all experiments where the irradiance was 47 μmol photons m^−2^ s^−1^ (during the light cycle). The algal strain in its exponential phase of growth served as inoculum and a 2 mL of homogenized culture was inoculated into 100 mL culture medium in 250 mL Erlenmeyer flasks to perform the time-series analyses. The experiments were conducted separately at three different temperatures (30, 40 and 50 °C) with triplicates for each treatment as well as for each time interval to enable destructive sampling on the days of observation.

### 4.4. Growth and physiological analyses

At periodic intervals, the growth and physiological parameters of the algal strain were monitored by following the standard methods. The total chlorophyll content was estimated following the method of MacKinney using a Beckman DU-64 spectrophotometer [28]. Carotenoids were extracted with 85% acetone as described by Jensen while the total proteins were estimated following the method of Lowry *et al*. [29,30]. Total and intracellular carbohydrates were measured following phenol-sulphuric acid method suggested by Dubois *et al*. [31]. Biomass, as total dry weight, was determined by filtering 25 mL of culture onto pre-weighed 4.7 cm Whatman GF/C glass fiber filters. The filters were then washed with deionised water to remove excess salts and dried in an oven at 90°C for 4 hours and then placed in a vacuum desiccator over silica gel and weighed. All amounts have been reported as μg per mL of the culture. Exponential regression of the logarithmic portion of the growth curve in terms of biomass and total chlorophyll were used to calculate the maximum specific growth rate (μ_max_, d^−1^).

The uptake of NaH^14^CO_3_ (specific activity 0.003 μCi μmol^−1^, Bhabha Atomic Research Centre, Mumbai) by algal strain as the end-point study was estimated after Kumar *et al*. [32]. Briefly, a known amount of homogenized culture suspension, pre-incubated in dark for 2 h, was supplemented with 100 μL of NaH^14^CO_3_. The algal samples were allowed to photosynthesize for 30 min at saturating light intensities and the reaction was terminated by adding 100 μL of 37% formaldehyde. After centrifugation, the pellet and supernatant were separated and the unused radio-labeled bicarbonate was driven off by adding one mL of concentrated acetic acid and then bubbling air through the mixture. The pellet was dried at 60°C, suspended in 10 mL of scintillation cocktail in a scintillation vial and counted in a Beckman LS-6000 SC liquid scintillation counter. To calculate the unused NaH^14^CO_3_, one mL of the supernatant was taken in a scintillation vial and 10 mL of scintillation cocktail was added to it. The rate of ^14^CO_2_ uptake was expressed in DPM (disintegrations per minute) or μmol of CO_2_ NaH^14^CO_3_ fixed mg chl^−1^. The carbonic anhydrase activity was assayed following the method of Dixon *et al*., which was a modification of the electrometric method [33]. The cells were harvested by centrifugation at 4,000 g, washed once with 25 mM veronal buffer (pH 8.2) and resuspended in buffer containing 1 mM dithiothreitol. The cell extract was prepared by disrupting the cells by passing through a French pressure cell (Simoaminco French Cell Press) at 100 mPa and clarified by centrifugation at 20,000 g for 30 min. The reaction mixture contained 5 mL of 25 mM veronal buffer (pH 8.2) and up to 2 mL of cell or enzyme extract at 3°C. Saturated CO_2_ solution (4 mL) was injected into the mixture by syringe and the decrease in pH was followed with time. For the uncatalysed reaction, boiled enzyme extract was used. Enzyme units were calculated from the time taken to lower the pH from 8.2 to 7.2 using the formula, E. U. = 10 [(t_b_ / t_c_) – 1)], where t_b_ and t_c_ were the time taken by the uncatalysed and enzyme catalysed reactions, respectively.

### 4.5. Statistical analysis

Data were statistically analysed following the methods suggested by Gomez and Gomez [34]. Individual and combined effects of carbon dioxide, temperature and incubation period on each growth parameter were analysed and the critical difference (C.D. or least significant difference) at 5% level of significance was determined after analysis of variance. The value of critical difference at 5% for CO_2_ concentration x temperature x days is only presented in Tables and Figures.

## Figures and Tables

**Table 1:** Specific growth rate (based on biomass and chlorophyll content) and biomass productivity of *C. vulgaris*.

CO_2_ (%)	Temperature (°C)	Biomass (μ_biomass_d^–1^)	Chlorophyll (μ_chl_ d^–1^)	Biomass (% increase after 10 d)
0.036 (ambient)	30	0.128	0.148	879
	40	0.082	0.121	361
	50	NG	NG	NG

6 (elevated)	30	0.222	0.262	1846
	40	0.136	0.200	874
	50	0.065	0.106	379

NG: No Growth

**Table 2:** ^14^CO_2_ uptake and carbonic anhydrase activity of *C. vulgaris* under elevated CO_2_ and temperature. C.D. at 5% level [CO_2_ concentration x temperature] for ^14^CO_2_ uptake and carbonic anhydrase activity is 0.340 and 0.520, respectively.

CO_2_ (%)	Temperature (°C)	^14^CO_2_ uptake (μmol NaH^14^CO_3_. mg chl^–1^ h^–1^)	Carbonic anhydrase activity (Enzyme units. mg chl^–1^)
0.036 (ambient)	30	7.833	14.811
	40	9.648	12.229
	50	n.d.	n.d.

6 (elevated)	30	6.033	7.861
	40	3.413	6.043
	50	3.086	5.893

## References

[1] Kondili EM, Kaldellis JK (2007). Biofuel implementation in East Europe: Current status and future prospects. Renew. Sust. Energ. Rev.

[2] Roman-Leshkov Y, Barrett CJ, Liu ZY, Dumesic JA (2007). Production of dimethylfuran for liquid fuels from biomass-derived carbohydrates. Nature.

[3] Gutierrez R, Gutierrez-Sanchez R, Nafidi A (2008). Trend analysis using nonhomogeneous stochastic diffusion processes. Emission of CO_2_; Kyoto protocol in Spain. Stoch. Environ. Res. Risk Assess.

[4] Wang B, Li Y, Wu N, Lan CQ (2008). CO_2_ bio-mitigation using microalgae. Appl. Microbiol. Biotechnol.

[5] de Morais MG, Costa JAV (2007). Biofixation of carbon dioxide by *Spirulina* sp. and *Scenedesmus obliquus* cultivated in a three-stage serial tubular photobioreactor. J. Biotechnol.

[6] Skjanes K, Lindblad P, Muller J (2007). BioCO_2_- a multidisciplinary, biological approach using solar energy to capture CO_2_ while producing H_2_ and high value products. Biomol. Eng.

[7] Li Y, Horsman M, Wu N, Lan CQ, Dubois-Calero N (2008). Biofuels from microalgae. Biotech. Prog.

[8] Hsueh HT, Chu H, Yu ST (2007). A batch study on the bio-fixation of carbon dioxide in the absorbed solution from a chemical wet scrubber by hot spring and marine algae. Chemosphere.

[9] Dismukes GC, Carrieri D, Bennette N, Ananyev GM, Posewitz MC (2008). Aquatic phototrophs: Efficient alternatives to land-based crops for biofuels. Curr. Opin. Biotechnol.

[10] Kodama M, Ikemoto H, Miyachi S (1993). A new species of highly CO_2_-tolerant fast growing marine microalga suitable for high density culture. J. Mar. Biotechnol.

[11] Miyachi S, Iwasaki I, Shiraiwa Y (2003). Historical perspective on microalgal and cyanobacterial acclimation to low- and extremely high-CO_2_ conditions. Photosynth. Res.

[12] de Morais MG, Costa JAV (2007). Isolation and selection of microalgae from coal fired thermoelectric power plant for biofixation of carbon dioxide. Energy Conv. Manag.

[13] Papazi A, Makridis P, Divanach P, Kotzabasis K (2008). Bioenergetic changes in the microalgal photosynthetic apparatus by extremely high CO_2_ concentrations induce an intense biomass production. Physiol. Plant.

[14] Xia JR, Gao KS (2005). Impacts of elevated CO_2_ concentration on biochemical composition, carbonic anhydrase and nitrate reductase activity of freshwater green algae. J. Integr. Plant Biol.

[15] Chiu SY, Kao CY, Tsai MT, Ong SC, Chen CH, Lin CS (2009). Lipid accumulation and CO_2_ utilization of *Nannochloropsis oculata* in response to CO_2_ aeration. Bioresour. Technol.

[16] Sakai N, Sakamoto Y, Kishimoto N, Chihara M, Karube I (1995). *Chlorella* strains from hot springs tolerant to high temperature and high CO_2_. Energy Conv. Manag.

[17] Mendes RL, Nobre BP, Cardoso MT, Pereira AP, Palavra AF (2003). Supercritical carbon dioxide extraction of compounds with pharmaceutical importance from microalgae. Inorg. Chim. Acta.

[18] Moroney JV, Husic HD, Tolbert NE (1985). Effects of carbonic anhydrase inhibitors on inorganic carbon accumulation by *Chlamydomonas reinhardtii*. Plant J.

[19] Moroney JV, Somanchi A (1999). How do algae concentrate CO_2_ to increase the efficiency of photosynthetic carbon fixation?. Plant Physiol.

[20] Hanagata N, Takeuchi T, Fukuju Y, Barnes DJ, Karube I (1992). Tolerance of microalgae to high CO_2_ and high temperature. Phytochemistry.

[21] DeLucia EH, Sasek TW, Strain BR (1985). Photosynthetic inhibition after long term exposure to elevated levels of atmospheric carbon dioxide. Photosynth. Res.

[22] Badger MR, Price GD (1994). The CO_2_ concentrating mechanism in cyanobacteria and green algae. Physiol. Plant.

[23] FitzGerald GP, Rohlich GA (1962). Biological removal of nutrients from treated sewage: Laboratory experiments. Verh Int Verein Theor Angew Limol.

[24] Travieso L, Pellón A, Benítez F, Sánchez E, Borja R, O’Farrill N, Weiland P (2002). BIOALGA reactor: preliminary studies for heavy metals metal. Biochem. Eng. J.

[25] Bhatnagar A (1999). Development of r- and K-selection model in the waste stabilisation pond system. J. Environ. Biol.

[26] Stanier RV, Kunisawa R, Mandel M, Cohen-Bazire G (1971). Purification and properties of unicellular blue-green algae (order: Chrococcales). Bacteriol. Rev.

[27] Rogers HH, Heck WW, Heagle AS (1983). A field technique for the study of plant responses to elevated CO_2_ concentration. Air Pollut. Control Assoc. J.

[28] MacKinney G (1941). Absorption of light by chlorophyll solutions. J. Biol. Chem.

[29] Jensen A, Hellebust JA, Craige JS (1978). Chlorophylls and carotenoids. Handbook of Physiological Methods.

[30] Lowry OH, Rosebrough NJ, Farr AL, Randall RJ (1951). Protein measurement with Folin-Phenol reagent. J. Biol. Chem.

[31] Dubois M, Gilles KA, Hamliton JK, Rebers PA, Smith F (1956). Colorimetric method for determination of sugars and related substances. Anal. Chem.

[32] Kumar A, Tabita FR, Van Baalen C (1982). Isolation and characterization of heterocysts from *Anabaena* sp. Strain CA. Arch. Microbiol.

[33] Dixon GK, Patel BN, Merrett MJ (1987). Role of intracellular carbonic anhydrase in inorganic carbon assimilation by *Porphyridium purpureum*. Planta.

[34] Gomez KA, Gomez AA (1984). Statistical procedures for agricultural research.

